# Experimental study of a 3D-printing technique combined with biphasic calcium phosphates to treat osteonecrosis of the femoral head in a canine model

**DOI:** 10.1186/s13018-023-04185-7

**Published:** 2023-09-16

**Authors:** Zhian Chen, Fanzhe Feng, Xixiong Su, Yongqing Xu, Ying Zhang, Hongbo Tan

**Affiliations:** 1 Graduate School, Kunming Medical University, Kunming City, Yunnan Province China; 2 Department of Orthopaedics, People’s Liberation Army Joint Logistic Support Force 920th Hospital, Kunming City, Yunnan Province China

**Keywords:** 3D-printing technology, Calcium phosphate ceramic, Osteonecrosis of the femoral head, Navigation template

## Abstract

**Objective:**

This study was aimed to use a digital design of 3D-printing technology to create a surgical navigation template. At the same time, biphasic calcium phosphate (BCP) was applied to treat osteonecrosis of the femoral head (ONFH) in animal models, based on accurate positioning of necrotic lesions in the navigation templates and observation of its therapeutic effect.

**Methods:**

Fifteen healthy adult male and female beagle dogs weighing 20 + 2 kg were randomly divided into three groups (*n* = 5) after establishing a model of ONFH using the liquid nitrogen freezing method. Each model underwent necrotic lesion creation and BPC implantations on one side of the femoral head and only necrotic lesion creation on the other side of the femoral head. Each group underwent CT examination, gross observation, histological examination and immunohistochemical staining at 6 weeks, 12 weeks and 18 weeks postoperatively.

**Results:**

At weeks 6, 12, and 18, CT and gross examination showed that the necrotic area in the experimental group was basically intact and had been completely raised by BCP material. In the control group, there were signs of bone repair in the femoral head, but there were still large bone defects and cavities. At week 18, extensive collapse of the cartilage surface was observed. Through histological examination, in the experimental group at 12 and 18 weeks, a large number of new and reconstructed bone trabeculae containing a large amount of collagen fibres were observed (*P* < 0.05), while in the control group, there was extensive necrosis of the bone trabeculae without cellular structural areas. Immunohistochemical examination observation: A large number of CD31-positive cells were observed in the experimental group at 6 weeks, gradually decreasing at 12 and 18 weeks (*P* < 0.05), while a small number of CD31-positive cells were observed in the control group at 18 weeks.

**Conclusion:**

The 3D-printed navigation template can accurately locate ONFH lesions. Implantation of BCP material can effectively play a supporting role, prevent the collapse of the loading surface, and induce bone formation and angiogenesis to some extent.

## Introduction

ONFH is a common and persistent disease in clinical practice. Researchers have found that it mainly occurs in men aged 30–50 years [1, 2]. ONFH is caused by traumatic fractures (femoral neck fractures, hip dislocation, severe hip sprain or contusion, hip surgery, etc.) and nontraumatic factors (long-term use of glucocorticoids, alcohol, sickle cell anaemia, AIDS, decompression diseases, etc.) [3–5]. These factors lead to a and hypoxia in the femoral head tissue, followed by necrosis of bone marrow and bone cells, progressive destruction of subchondral bone, difficulty in the self-repair process of cellular tissue, and ultimately joint collapse and degenerative arthritis [6, 7]. The clinical symptoms of avascular necrosis of the femoral head are relatively hidden, and patients usually seek medical attention when they feel pain in the hip joint. By then, the disease has often progressed to ARCO stage II or even ARCO stage III established by the International Association Research Circulation Osseous (ARCO) [8]. At this time, nonsurgical repair of femoral head necrosis is more difficult, so how to effectively treat ONFH has long been a major challenge faced by doctors.

Currently, the commonly used surgical methods for the treatment of femoral head necrosis include core decompression, vascularized bone grafting, osteotomy, and joint replacement [9–11]. Although these methods have achieved great success in clinical applications, with the increasing health demand, these surgical methods have shown many inconveniences. The long-term efficacy is poor due to factors such as nutritional deficiencies in the necrotic area, insufficient oxygen supply, and difficulty in vascular reconstruction after decompression surgery [11, 12]. Autologous vascularized bone flap transplantation requires sacrificing healthy tissue, which falls in the category of "robbing the east wall to pay the west wall" and causes secondary injuries. Artificial hip joints, on the other hand, exist in the form of foreign objects in the body, which limits their performance and lifespan. In the later stages, patients will need to undergo further or even multiple revisions, seriously affecting their quality of life and causing long-term pain and distress to both the patients and their families [13–15]. At the same time, the development of biomaterials has entered a new stage, and the discovery and confirmation of bone-induced properties of biomaterials have provided new avenues for the concept of "repair, reconstruction, and regeneration" advocated by bone science in the new era. BCP has attracted researchers' interest due to its hydroxyapatite (HA) and b-tricalcium phosphate (β-TCP) mixed composition [16], which has good biocompatibility, bioabsorbability, biological activity, and osteogenic and angiogenic abilities and has been widely used in bone tissue, periodontal and dental tissue repair and has achieved good therapeutic effects [17–21]. Previous studies have shown that the chemical composition of a 60% HA/40% β-TCP mixture is close to that of bone, with the strongest impact on fracture regeneration ability and appropriate mechanical properties [22]. Therefore, the repair effect of BCP composed of 60% HA/40% β-TCP on femoral head necrosis is worth studying.

In this study, we attempted to use 3D-printed navigation templates to accurately locate necrotic lesions and applied BCP materials to the treatment of ONFH in animal models. We focused on exploring the short-term performance and effectiveness of BCP materials in ONFH treatment to accumulate more data for the preparation and clinical application of future bone-inducing biomaterials.

## Materials and methods

### Experimental animals and materials

This study used 15 mature adult dogs without femoral head injury (Chengdu Dashuo Biotechnology Co., Ltd., China), regardless of sex, with an average weight of 20 ± 2 kg and an average age of 16 ± 1 month. Standard animal laboratory dog feed, room temperature 20–25 °C, relative humidity 50–70%, regular drinking water and lighting were provided. The experimental environment met the relevant standards set by the International Society for the Protection of Animals, as well as the necessary conditions for the life of beagles. During the experiment, strict adherence to humanitarian norms and requirements was maintained, and the disposal of the animals met humanitarian requirements. This study was approved by the Ethics Committee of Hospital 920 of the Joint Logistics Support Force (No. 2017-056 (Department) -01).

### Study design

Before surgery, a 3D-printing template was designed based on the hip joint of beagles, and a femoral head necrosis model was established using liquid nitrogen freezing [23, 24]. Fifteen beagles with bilateral femoral head necrosis were randomly assigned, with one femoral head undergoing necrotic lesion removal and BCP material implantation as the experimental group and the other femoral head undergoing only necrotic lesion removal as the control group. Five beagles were randomly selected and killed at 6 weeks, 12 weeks, and 18 weeks after surgery. Femoral head specimens were taken for macroscopic observation of the defect repair tissue, CT scanning for evaluation, and histological and immunohistochemical evaluation.

### 3D printed navigation template preparation

**Fig. 1.**
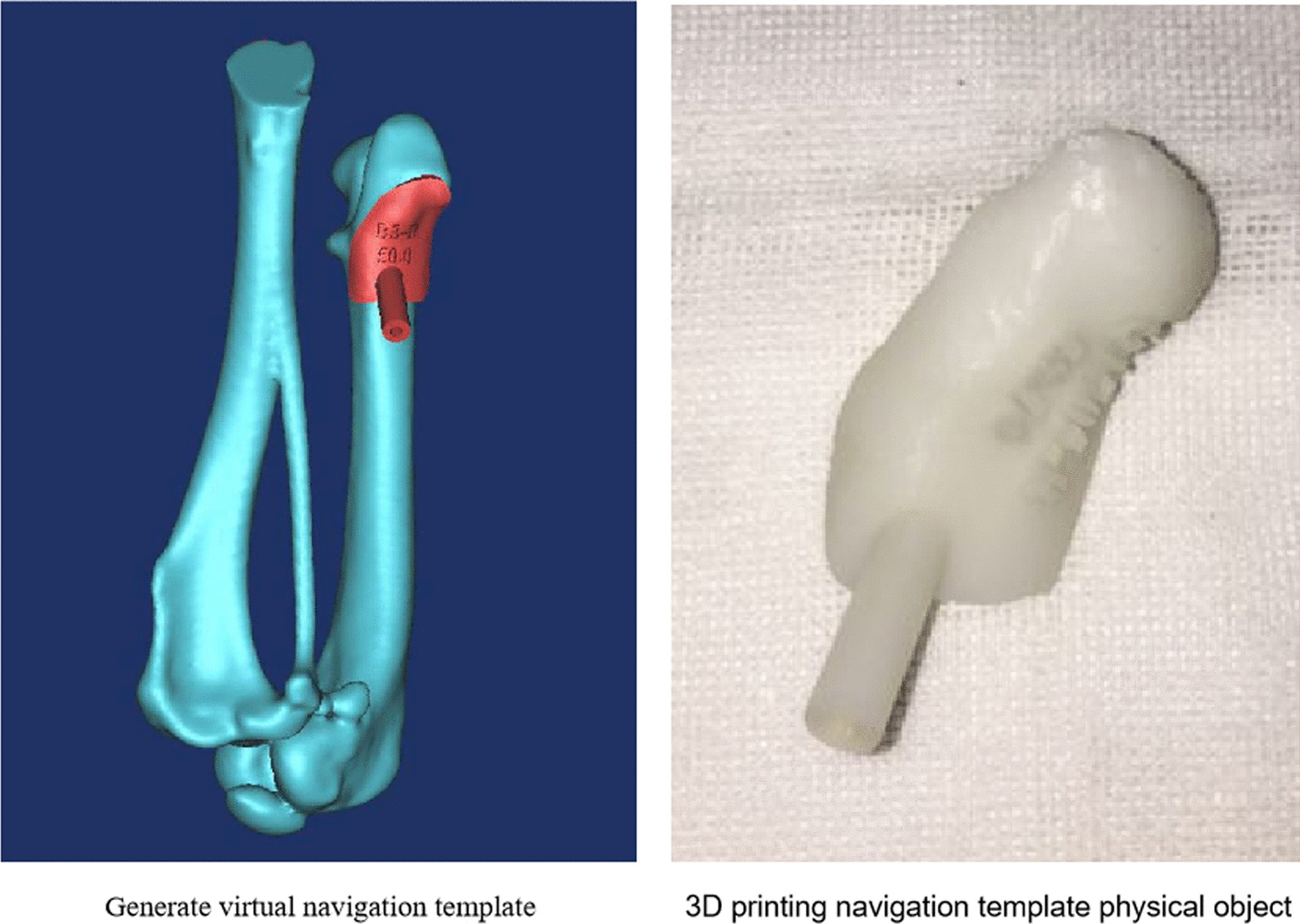
3D-printing navigation template production

The beagle dogs were randomly selected, and 3% pentobarbital sodium solution was used for intravenous anaesthesia (1 ml/kg, Merck Chemical Technology Co., Ltd., China). After stable anaesthesia, the dogs were fixed in the prone position on the 64-row spiral CT (GE Medical Systems, USA) platform to conduct CT thin slice scanning of each beagle dog's bilateral hip joints, and the original CT image data were imported into Mimics 15.0 software (Materialise Corporation, Belgium) in the DICOM format. In Mimics 15.0 software, the three-dimensional model of the femoral head was reconstructed through functions such as threshold segmentation, region growth, Boolean operation, and mask smoothing. The navigation tube part of the template was designed using the MedCAD module of Mimics 15.0 software, the optimal needle insertion angle and orientation were determined, and the apex of the navigation tube was ensured to reach the weight-bearing area below the cartilage surface of the canine femoral head. Using the greater trochanter region of the canine femoral head as the template domain, a positioning template with the same surface bone anatomical structure as the template domain was reverse established, and a navigation tube was placed into the positioning template. The STL data were imported into Geomagic Studio 12.0 software (Geomagic Corporation, USA), the extracted positioning template was accurately matched with the navigation tube, and a virtual navigation template was digitally generated in STL format. The virtual navigation template data in STL format were imported into the SPS350B solid laser rapid prototyping machine (Shaanxi Hengtong Intelligent Machine Co., Ltd., China), and the physical navigation template was made of photosensitive resin 14120 (DSM Somos Corporation, USA) and stereolithography apparatus (SLA). The related machine parameter settings were as follows: Processing layer thickness: 0.1 mm; Processing accuracy: 0.1 mm; Laser scanning speed: 10 m/s; Power: 3 kW. Residual support was removed from the template, and photocuring treatment was performed. Low-temperature plasma disinfection was performed on the template before surgery (Fig. 1).

### Preparation of BCP materials

BCP (Sichuan University Biomaterials Research and Engineering Center, China) powder is composed of HA/β-TCP phosphate, which is synthesized by the wet coprecipitation method in the proportion of 60% HA/40% β-TCP. The BCP material produced was sterilized by radiation for use.

### Surgical methods

The double hips of the beagle dog were randomly selected and labelled as the experimental group using a computer. After successful anaesthesia, the dog was placed in a lateral lying position, the hair was removed, and skin on the surgical area of the rear buttocks was prepared and disinfected. Towels were spread routinely. An arc incision was made through the outer side of the hip joint, approximately 7 cm long, to separate the soft tissue and expose the proximal femur and greater trochanter. The soft tissue attached to the large rotor and its surrounding area was peeled off, and the 3D printed navigation template was attached to the corresponding bone surface according to the preoperative design. Using an electric drill (Smith&Nephew Corporation, USA), a prelabelled 1.5 mm diameter Kirschner wire (Shanghai Medical Device Group Co., Ltd., China) was inserted through the navigation hole of the 3D printed navigation template. The 3D-printed navigation template was removed, and a prelabelled 4.0 mm diameter hollow drill was inserted along the Kirschner wire into the femoral head using an electric drill to establish a bone tunnel that reached the predetermined necrotic area. During the surgery, a C-arm X-ray machine (SIMENS Healthineers Corporation, USA) was used for fluoroscopy to determine the arrival of the bone tunnel at the predetermined area of the femoral head. Gauze was used to protect surrounding soft tissues, and a cotton swab was dipped in liquid nitrogen (Kunming Medical Oxygen Plant, China), quickly extended into the top of the bone tunnel, and removed after standing for 30 s. A cotton swab was dipped in 50 °C physiological saline (Kunming Nantian Chemical Pharmaceutical Co., Ltd., China), quickly extended into the top of the bone tunnel, and allowed to stand for 30 s to warm it up again. The above process of freezing in liquid nitrogen for 30 s and rewarming in 50 °C physiological saline for 30 s was repeated 3 times. After using a C-arm X-ray machine to establish the model, the drill bit was repeatedly polished, and the necrotic lesion and surrounding hardened bone were removed. Physiological saline was used to wash the wound, and satisfactory blood leakage was observed on the polished wound surface of the femoral head, indicating that the necrotic area of the femoral head was thoroughly cleared. According to the established bone tunnel size and depth, the experimental group of beagles selected an appropriate size of BCP material. First, an appropriate amount of granular BCP material (φO = 3 mm) was carefully placed along the bone tunnel, and a bone grafting device was used to moderately compress the BCP material to make it fit tightly. If the implantation amount was insufficient, granular BCP material was repeatedly added and compacted. The control group did not receive BCP material implantation. The dressing was changed daily after surgery, rinsed and disinfected with iodine and physiological saline, and wrapped with dressing until the incision was completely healed.

## Evaluation indicators

### Imaging examination

After execution, the pigs were immediately sent to the 920th Hospital of Joint Logistics Support Force for computed tomography (CT) examination. Scan data settings: voltage 120 kV, current 150 MA, matrix 512 * 512, layer thickness 0.625 mm. CT examination of the implanted BCP material area for femoral head necrosis, evaluation of bone repair, tissue structure repair, and the presence of subchondral bone cysts.

### Gross evaluation

Pentobarbital (0.4 mL/kg) was injected intravenously to euthanize the beagle dogs 6 weeks, 12 weeks and 18 weeks after the operation. Bilateral femoral head joint samples were collected. The femoral head was observed along the plane where the neck shaft angle was located, and the appearance, necrosis, and BCP material implantation and repair of the femoral head were observed with the naked eye.

### Histological examination

The tissue was fixed with polyoxymethylene (4% PFA) for 48 h, and decalcifying solution was prepared with 5% concentrated hydrochloric acid (Kunming Nantian Chemical Pharmaceutical Co., Ltd., China), 5% concentrated formic acid (Kunming Nantian Chemical Pharmaceutical Co., Ltd., China), and 75% alcohol (90%). The femoral head specimens were decalcified for 48 h. After the decalcification of the test sample was completed, it was dehydrated through a graded alcohol series, cleared with xylene, soaked in wax, and then embedded. The paraffin was fixed on the slicing machine, the thickness of the slice on the slicing machine was adjusted to 4 µm, and paraffin slicing was performed, with dewaxing to water. Staining was performed using a haematoxylin eosin staining kit (G1120, Solarbio, Shanghai, China) and Masson's staining kit (G1340, Solarbio, Shanghai, China). An Olympus CX33 microscope (Olympus, Japan) was used for microscopy, image acquisition and analysis.

### Immunohistochemical staining

After decalcification, samples were dehydrated through a graded alcohol series, cleared with xylene, soaked in wax, and then embedded. The paraffin was placed on a microtome to fix it, the slice thickness on the microtome was adjusted to 4 µm, and paraffin sections were performed, with routine deparaffinization to water. Sodium citrate antigen retrieval (C1031, Solarbio, Shanghai, China) was followed by natural cooling and incubation in 3% H2O2 deionized water for 10–30 min. After blocking with 10% goat serum (SL038, Solarbio, Shanghai, China) for 5–30 min, the anti-CD31 primary antibody (ER31219, rabbit polyclonal antibody, HuaBio, Guangzhou, China) was added overnight at 4 °C. A ready-to-use immunohistochemical ultrasensitive s-p hypersensitivity reagent (MXB Maixin reagent, KIT-9706) was added dropwise, and DAB solution was used for color development (DA1016, Solarbio, Shanghai, China). The slides were rinsed with distilled water, counterstained with haematoxylin, dehydrated, cleared, and sealed. An Olympus CX33 microscope (Olympus, Japan) was used for microscopy, image acquisition and analysis.

### Statistics analysis

All results are expressed herein as the mean ± standard deviation (IBM SPSS Statistics 23.0 statistical software). Due to the need to evaluate the differences between two sets of individual indicators, paired tests were conducted on normal data. If the data did not meet the normal distribution, logarithmic transformation was performed, and then the data were reanalyzed. ImageJ (version 1.8.0.345) was used to measure data on collagen fibres and trabeculae. All data were scored using GraphPad Prism (version 9.0). A value of *P* < 0.05 was considered statistically significant.

## Results

### Comparative observation of imaging

**Fig. 2:**
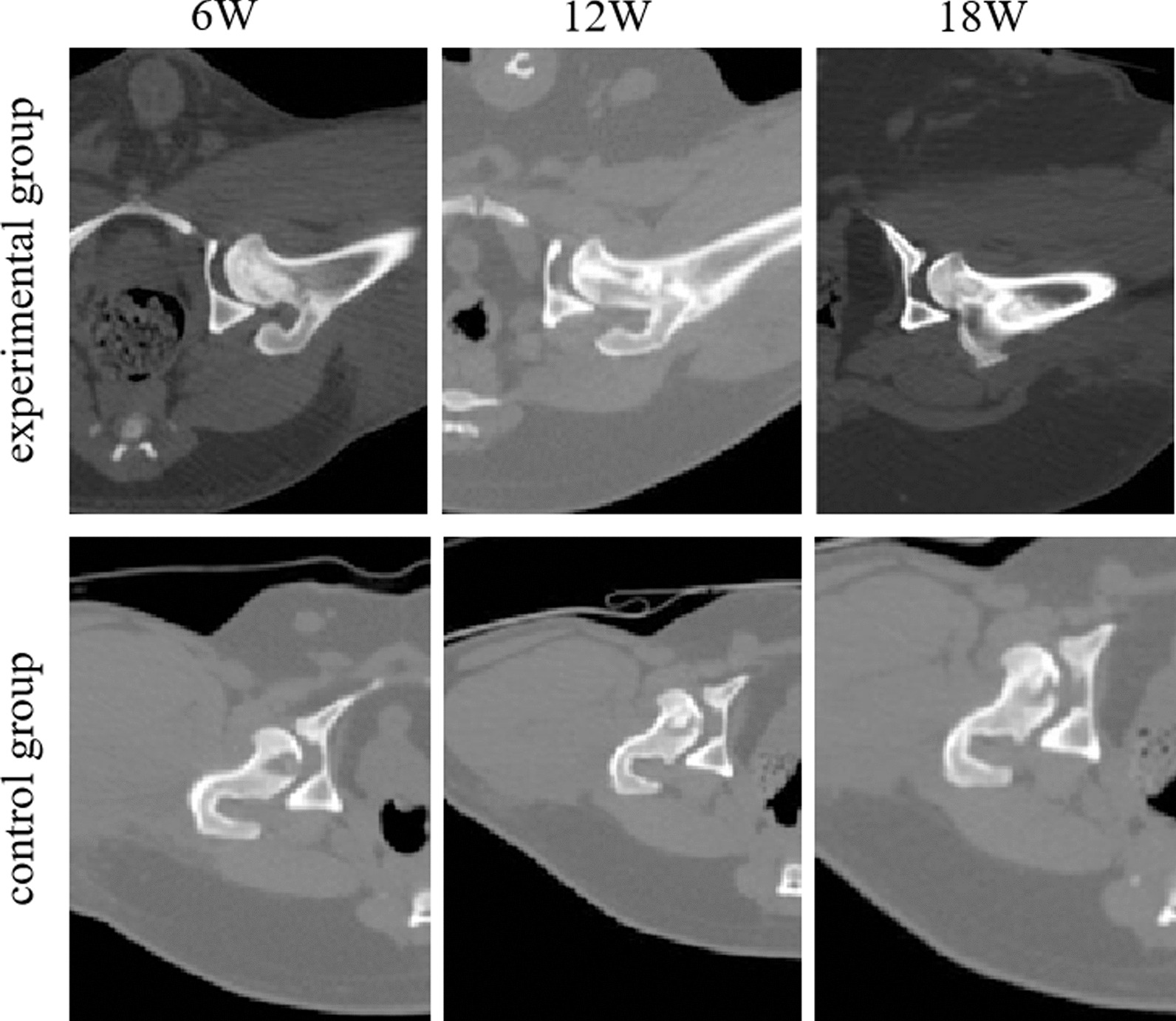
Example sagittal and coronal CT images of the experimental group and control group at 6 W, 12 W, and 18 W after the operation

The imaging examination showed that the implanted BCP material in the experimental group was in an objective position, and its structure was basically complete and columnar. Compared to that in the control group, the collapsed area of femoral head necrosis had been completely lifted, and the BCP material implanted at weeks 12 and 18 showed significant absorption compared to that at week 6. In the control group, the necrotic bone in the femoral neck area had been removed. At 6 weeks, cavity formation was observed in the defect area, and at 12 and 18 weeks, a small amount of bone tissue was repaired in the necrotic area, but there was still a cavity in the bone defect area (Fig. 2).

### Gross observation and section observation

**Fig. 3:**
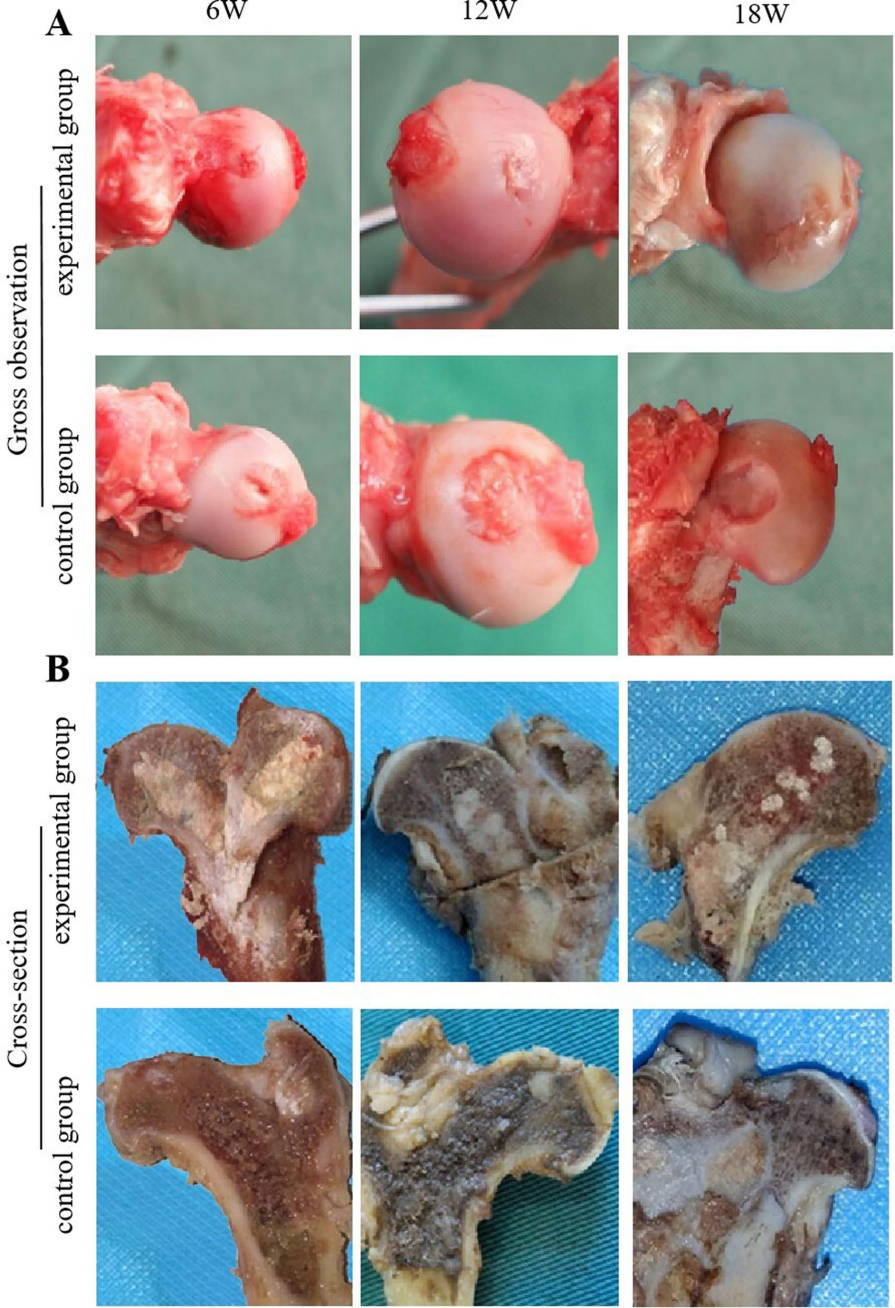
**A** Gross observation of the femoral head in the experimental group and control group: 6 W, 12 W, and 18 W after the operation; **B** Cross-section of the femoral head in the experimental group and control group: 6 W, 12 W, and 18 W after the operation

Overall observation showed that at 6 weeks, the experimental group showed implanted BCP material, and the cartilage on the tunnel surface began to be repaired. Compared with that in the control group, the femoral head and cartilage surface structure were more complete, with slight damage to the cartilage surface. At 12 weeks, the experimental group showed a large amount of cartilage repair on the surface of the tunnel, while the control group showed a gradual increase in cartilage surface damage. At 18 weeks, the experimental group showed that the cartilage on the surface of the tunnel had been basically repaired, while the control group showed necrosis and fragmentation of the cartilage surface of the femoral head in a circular shape, with an inwards collapse area visible in the middle (Fig. 3A).

The cross-sectional observation of the femur showed that the BCP material implanted in the experimental group was visible to the naked eye and structurally intact. At weeks 12 and 18, a small amount of BCP material was absorbed and replaced by bone tissue. Compared with that in the control group, the femoral head was circular, with a more complete structure of the medullary cavity and cartilage surface. The femoral head in the control group was elliptical in shape, with incomplete and fragmented cartilage surfaces in the load-bearing area. Some cartilage in the middle area was visible to the naked eye and collapsed inwards into the medulla (Fig. 3B).

### Histologic results

**Fig. 4:**
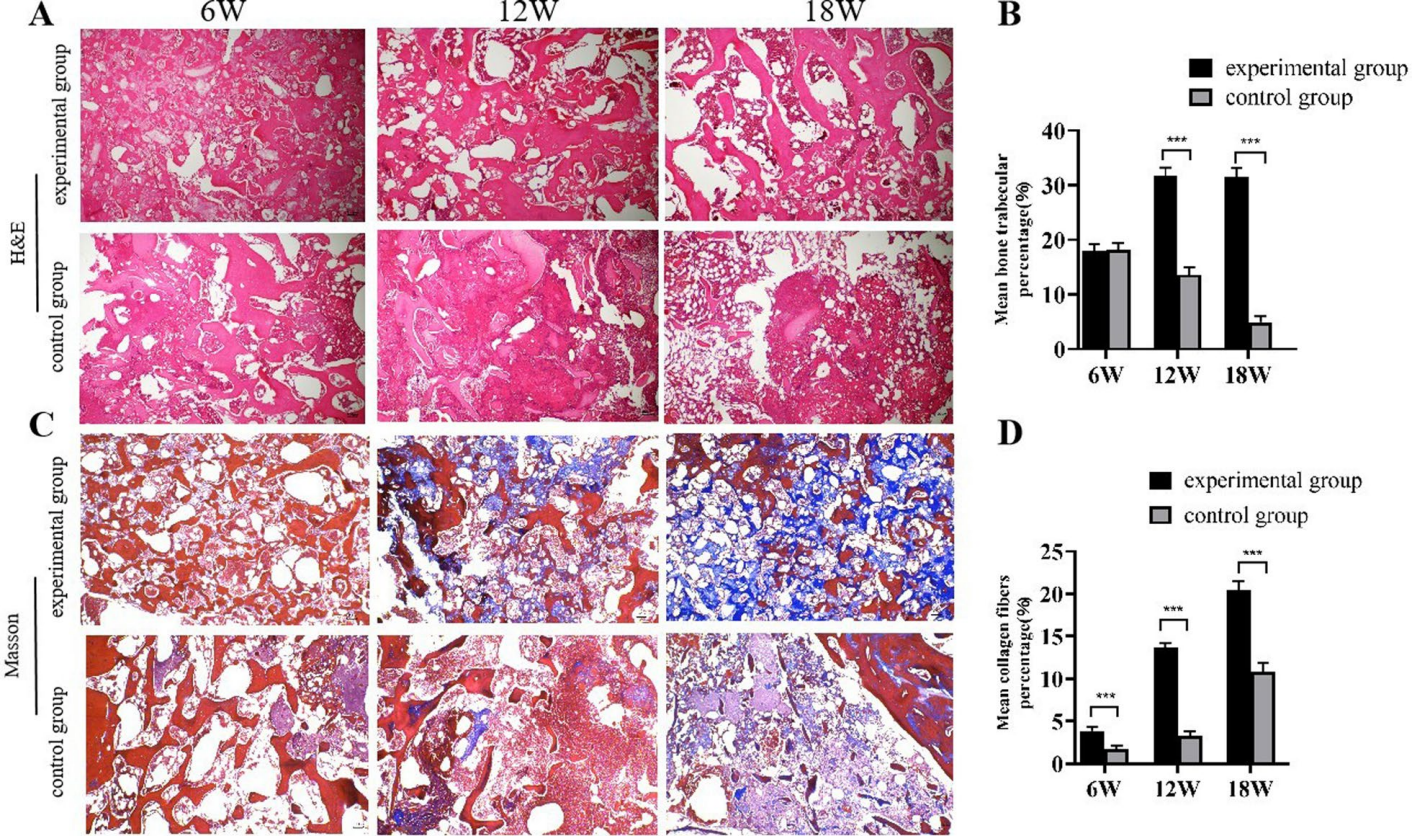
**A** H&E staining of the femoral head in the experimental group and control group: 6 W, 12 W, and 18 W after the operation; **B** Quantitative results showing the percentage of bone trabeculae in H&E staining between groups; **C** Masson staining of the femoral head in the experimental group and control group: 6 W, 12 W, and 18 W after the operation; **D** Quantitative results showing the percentage of collagen fibres in the Masson trichrome staining between groups. Scale bar = 100 µm. Asterisks indicate statistical significance (*p < 0.05, **p < 0.01, ***p < 0.005)

We further conducted histological H&E staining (Fig. 4A). At 6 weeks, the femoral slices from the experimental group showed a large amount of BCP material that was colorless, honeycomb-shaped, and tightly arranged and that filled the necrotic area of the femoral head. At 12 and 18 weeks, the BCP material gradually degraded and was replaced with bone tissue. In the experimental group, a small amount of new bone trabeculae appeared at 6 weeks, slightly stained red, connected to the original bone trabeculae, and the vascular sinuses were filled with blood. At 6 weeks of femoral sectioning in the control group, the bone trabeculae were sparsely fractured. After 12 weeks, adipocytes proliferated and merged into a large cavity, presenting a purple necrotic area without acellular structure. At 18 weeks, the necrotic area gradually expanded. The quantitative results (Fig. 4B) showed that the percentage of bone trabeculae in the experimental group at 12 and 18 weeks was higher than that in the control group, and the difference was statistically significant (p < 0.05).

We further performed Masson staining (Fig. 4C). Masson staining was mainly red in bone tissue, with blue staining representing collagen fibres. Compared with the control group, the experimental group showed a large number of blue collagen fibres, and collagen gradually increased from 6 to 18 weeks. The control group showed sporadic collagen positivity without obvious stripes. The quantitative results (Fig. 4D) showed that the percentage of collagen fibres in the experimental group at weeks 6, 12, and 18 was higher than that in the control group, with a statistically significant difference (*p* < 0.05).

### Immunohistochemical staining

**Fig. 5:**
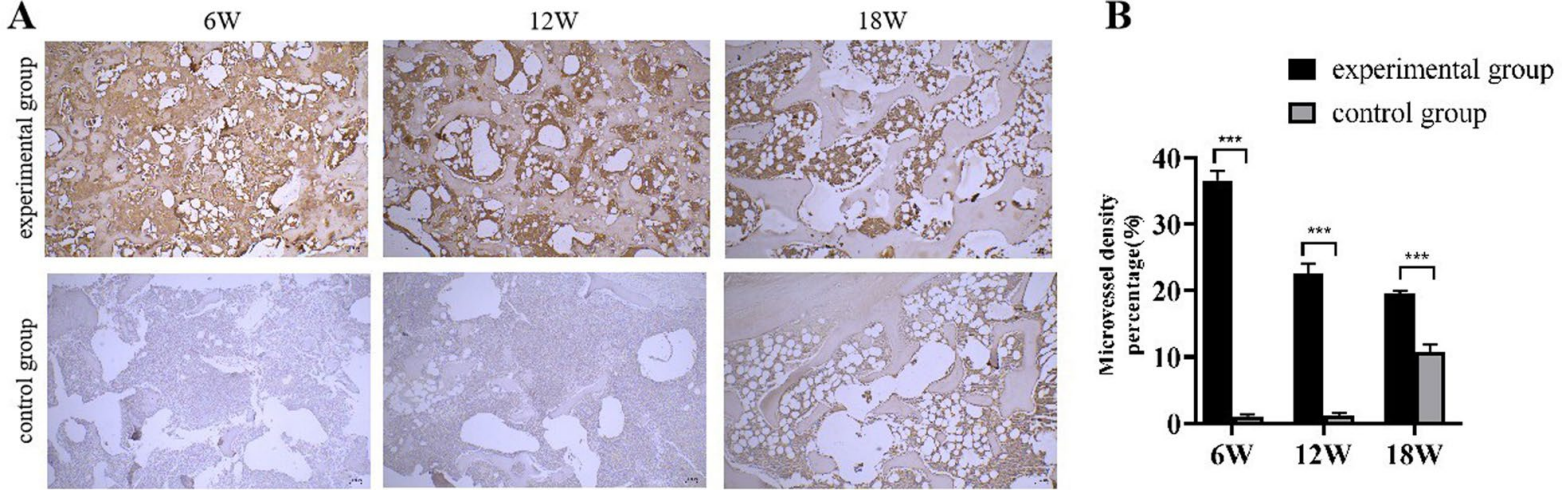
**A** IHC staining of the experimental group and control group: expression of CD31 at 6 W, 12 W, and 18 W; **B** Quantification of CD31 expression level. Scale = 100 µm. The asterisk indicates statistical significance (*p < 0.05, **p < 0.01, ***p < 0.005)

We used immunohistochemistry to evaluate the expression of the angiogenic marker CD31 (Fig. 5A). In the experimental group, a large number of CD31-positive cells were observed at 6 weeks, and over time, the number of CD31-positive cells gradually decreased at 12 and 18 weeks. The control group showed no significant changes in CD31-positive cells at 6 and 12 weeks, while a small number of CD31-positive cells were observed at 18 weeks. The quantitative analysis results of CD31 (Fig. 5B) showed significant differences between the control group and the experimental group at 6, 12, and 18 weeks.

## Discussion

The main finding of this study is that on the 3D-printed navigation template to create a precise model of femoral head necrosis, the experimental group showed obvious placement of BCP material through CT and gross observation, and the structure of the femoral head and cartilage surface was relatively complete, without significant collapse. In addition, we confirmed through immunohistochemistry that the implanted BCP material showed obvious CD31-positive cells. Finally, our study also indicates that the use of beagles as a model for femoral head necrosis is reliable.

Research shows that ONFH is an ischaemic disease, and the current treatment focus is on the relief of patients' symptoms. Hip joint replacement is the only effective treatment method, but it is not applicable to young and middle-aged patients and has many disadvantages. Femoral head necrosis gradually worsens over time and presents necrotic areas, which are surrounded by irregular repair reactions such as granulation, fibrous tissue, and sclerotic cancellous bone [25]. As bone cells disappear, calcified bone marrow appears, bone density decreases, and femoral head collapse follows [26]. Bohndorf et al. [27] believe that early imaging of femoral head necrosis can reveal patchy osteoporosis, sclerosis, cysts, and crescent signs. However, in the femoral head necrosis model established by liquid nitrogen in this experiment, imaging revealed a large cavity at the bone defect in the blank group at 6 weeks. At 18 weeks of gross observation, the necrotic area collapsed, approximately located at the edge of the acetabulum. Ansari et al. [28] concluded that when the lesion area exceeds 25% of the femoral head area, 90.6% of the hip joint collapses within one year. In addition to the necrotic area, femoral head collapse is also affected by the lesion location. Several studies have shown that the collapse rate of lesions that exceed 2/3 of the inner side of the weight-bearing part is high. When the lesion extends laterally to the edge of the acetabulum, femoral head collapse is more common [29–31]. The experimental group implanted the BCP material in a prominent imaging position, with a basically intact structure and a columnar shape. Overall observation showed no collapse in the necrotic area of the femoral head. By imaging and gross observation, it can be concluded that the implantation of BCP material into hips with ONFH can effectively prevent the collapse of the necrotic area of the femoral head.

Calcium phosphate ceramics, as artificial bone substitute materials, have been widely used in various types of bone defect repair after long-term exploration and research, as reported by Nery et al. [32] in 1975. At present, there are three kinds of ideal artificial bone substitutes that are suitable for application: HA, β-TCP, and BCP [16, 33, 34]. Among them, HA has high strength and good support but a slow degradation rate; β-TCP has good degradation performance and osteogenic ability, but its strength is poor and it is prone to collapse under certain pressures [35]; β-TCP material is prepared from hydroxyapatite and β-TCP according to a certain calcium phosphorus ratio. The HA/β-TCP ratio mainly includes 15/85 [36], 20/80 [37], 25/75 [38], 30/70 [39], 50/50 [40] and 60/40 [22]. When the HA/β-TCP ratio is 60/40, it can promote cell proliferation to a greater extent. In animal experiments, Jensen et al. [16] divided the HA/β-TCP ratio into 60/40, 100/0, and 0/100 groups when studying mandibular bone defects in pigs. Through histological evaluation, it was found that the bone formation rate was fastest when the HA/β-TCP ratio was 60/40. However, Park et al. [41] compared 60/40 and 0/100 in a rabbit skull defect experiment and found that when the HA/β-TCP ratio was 60/40, the proportion of new bone with a faster biodegradation rate was the highest, which could be used as an effective bone conduction material for bone defects and showed the same effect in rat skull defects [42]. This experiment revealed through histological staining in a model of femoral head necrosis that BCP gradually degrades over time, while bone trabeculae and collagen fibres gradually increase, which has a positive therapeutic effect on femoral head necrosis.

Similar to other biomaterials, BCP has the ability to adsorb proteins, cells, and other biological organic molecules, which facilitates osteogenesis and angiogenesis in the defect area [43]. In terms of osteogenesis, BCP not only plays a supporting role but can also migrate bone marrow mesenchymal stem cells to the BCP implantation site through blood circulation to induce the differentiation of bone marrow mesenchymal stem cells [44]. At the same time, Li et al. [45] studied the bone induction and bone regeneration ability of BCP by implanting nanocrystalline porous BCP ceramic balls into the back muscles of Birgel dogs and the mandible of rabbits and found that nanocrystalline porous BCP ceramic balls could be a potential substitute for bone defect filling. Zhang et al. [46] studied the ossification of BCP with different apertures through coculture of BCP and BMSCs and implantation of BCP into the back muscles of Birgel dogs in animal experiments. They found that BCP materials with different apertures had different osteogenic abilities, and there were significant differences in the upregulation of genes related to the Notch pathway. The results showed that BCP materials activated the expression of genes related to osteogenesis through the Notch signaling pathway. By increasing the β-TCP/HA ratio, bone formation can also be enhanced, as it can enhance the biological absorption rate, thereby releasing more Ca and P ions and triggering osteogenic differentiation. The effect on angiogenesis is mainly due to the release of calcium ions. The higher the Ca ions, the stronger the angiogenesis l [17]. In the experiment, a large amount of angiogenesis was observed through immunohistochemical staining with the angiogenic marker CD31, which showed statistical significance compared to the experimental group. Over time, it was observed that CD31 gradually decreased in the experimental group, mainly due to the gradual degradation of BCP materials and osteogenic effects.

Through this group of experiments, we believe that the combination of digital design 3D-printing technology and BCP material has the following advantages in treating ONFH: (1) precise positioning, avoiding the occurrence of deviation from the lesion, shallow or deep needle insertion, and even penetrating the cartilage surface of the femoral head caused by empirical needle insertion in the past; (2) according to the template navigation hole, needle insertion reduces the difficulty of surgery and is suitable for young doctors to use; (3) calcium phosphate ceramics have good supporting performance, gradually begin to degrade after implantation, and can effectively induce osteogenesis and angiogenesis; and (4) the surgical wound is smaller, and the material cost is lower. At the same time, this technology also has some shortcomings, such as the slow bone induction speed of BCP materials in the short term, and it is necessary to explore how to adjust the calcium phosphorus ratio of BCP materials to optimally match the growth rate of new bone in the femoral head marrow. The sample size of this experiment is small, comparative research with various traditional techniques is lacking, and the postoperative observation time is short. It is necessary to increase the sample size and prolong the observation period to obtain more accurate experimental data.

In the current study, we demonstrated that BCP is an effective method for treating ONFH. The experimental group achieved certain results in the gross, imaging, histological, and immunohistochemical evaluations. Based on the findings of this study, we conclude that BCP can be used for the treatment of ONFH.

## Data Availability

All datasets presented in this study are included in the article.
